# Hydrodynamic trapping measures the interaction between membrane-associated molecules

**DOI:** 10.1038/s41598-018-30285-0

**Published:** 2018-08-20

**Authors:** Victoria Junghans, Jana Hladilkova, Ana Mafalda Santos, Mikael Lund, Simon J. Davis, Peter Jönsson

**Affiliations:** 1 Department of Chemistry, Lund University, SE-22100 Lund, Sweden; 2 Weatherall Institute of Molecular Medicine, University of Oxford, Oxford, OX3 9DS UK

## Abstract

How membrane proteins distribute and behave on the surface of cells depends on the molecules’ chemical potential. However, measuring this potential, and how it varies with protein-to-protein distance, has been challenging. Here, we present a method we call hydrodynamic trapping that can achieve this. Our method uses the focused liquid flow from a micropipette to locally accumulate molecules protruding above a lipid membrane. The chemical potential, as well as information about the dimensions of the studied molecule, are obtained by relating the degree of accumulation to the strength of the trap. We have used this method to study four representative proteins, with different height-to-width ratios and molecular properties; from globular streptavidin, to the rod-like immune cell proteins CD2, CD4 and CD45. The data we obtain illustrates how protein shape, glycosylation and flexibility influence the behaviour of membrane proteins, as well as underlining the general applicability of the method.

## Introduction

Both the interaction and the transfer of information between cells is regulated via membrane proteins acting, among other roles, as receptors, adhesion molecules, or ion transporters. Vital for this is the interaction with other membrane proteins. These interactions can be either permanent or transient, allowing for changes in the oligomeric state of the molecules^1^. However, little is known about the physicochemical forces acting between membrane-anchored molecules and previously it has been problematic to study the interactions between molecules bound to lipid bilayers. We recently showed that the liquid flow through a micropipette can be used to trap and accumulate molecules in a supported lipid bilayer (SLB), a technique we call hydrodynamic trapping^2,3^. The flow acts on molecules protruding from the lipid bilayer with a drag force whose magnitude can be accurately controlled by varying the flow rate through the pipette and distance to the SLB. It is in this way possible to change the local concentration of membrane-bound molecules by orders of magnitude^2^. Here we show how hydrodynamic trapping can be used to measure the chemical potential, and thus the force, between membrane-anchored molecules. Combined with Metropolis-Hastings Monte Carlo (MC) simulations, this allows us to draw a series of general conclusions regarding how protein shape, glycosylation and flexibility affect the interaction between membrane-associated molecules.

**Figure 1:**
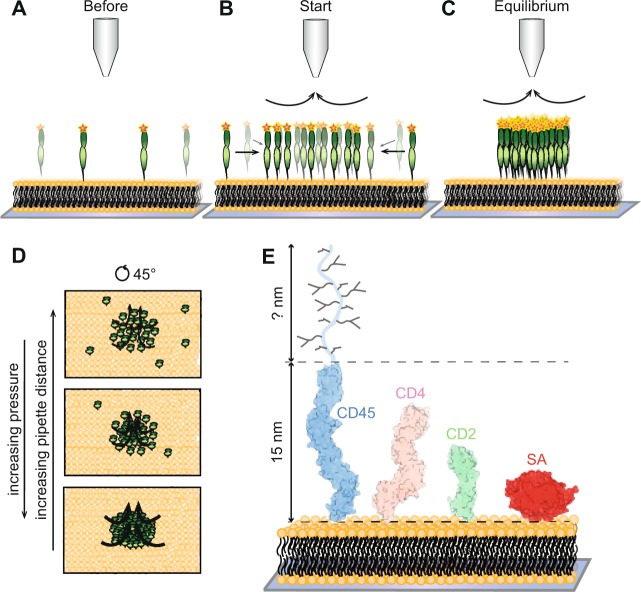
Schematic illustration of the experiments and the studied molecules. (**A**–**C**) Hydrodynamic trapping of membrane-anchored molecules in an SLB is achieved by applying negative pressure through a micropipette (tip radius ~ 1 μm). This results in accumulation of the molecules in the SLB. Not drawn to scale. (**D**) The accumulation depends on the applied pressure and the distance between the pipette and the SLB. (**E**) Dimensions of the studied molecules: human CD45 (CD45d1-d4: PDB 5FMV + mucin-like region), human CD4 (PDB 3T0E), rat CD2 (PDB 1HNG) and SA (PDB 3RY2).

The basis of the method is the following. A micropipette is positioned above an SLB containing one or two sorts of proteins, at an average surface density of 100 to 1000 molecules/µm^2^ (see Fig. 1A). Negative pressure is applied through the micropipette and the proteins start to accumulate (see Fig. 1B,C). The higher the applied pressure, and/or the closer the micropipette is to the surface, the higher is the force acting on the molecules, resulting in an increased protein accumulation (see Fig. 1D). We can estimate: (i) the approximate dimensions of the molecule, and (ii) the intermolecular forces between the molecules and how this varies with protein-to-protein distance, by relating the accumulation to the strength of the hydrodynamic trap.

We selected four membrane-anchored molecules of varying shape, level of glycosylation and flexibility to investigate with our method. These molecules were the B vitamin biotin-binding protein streptavidin (SA), and the immune-cell membrane proteins CD2, CD4 and CD45RABC (CD45). SA has a globular shape (width and height of ~5 nm)^4^ and is used in numerous biotechnological and diagnostic applications^5^. The three immune-cell proteins have, presumably, rod-like shapes, with a cross-sectional width of ~3 nm, but vary in height from approximately 7 nm to potentially over 40 nm, see Fig. 1E^6–9^. CD2, which is important for stable cell-cell adhesion between immune cells and is the smallest of these proteins (height ~7.5 nm)^6^, is also heavily glycosylated. CD4 (height ~11 nm) is known to interact with MHC class II molecules on antigen presenting cells (APCs), but with a very low affinity^8,10^, heightening T-cell sensitivity to foreign antigen. CD45 is a receptor type tyrosine phosphatase regulating T-cell signalling, suggested to be excluded from the close contact area formed between immune cells and their targets due to its large protein size and heavy glycosylation (height >15 nm)^7,11^. The orientation and interaction between these molecules are not only interesting from a modelling point of view, but will affect how these molecules distribute and behave in the contact between immune cells, which are key factors to better understand immune-cell signalling.

## Results

### From trapping to interaction curves

**Figure 2:**
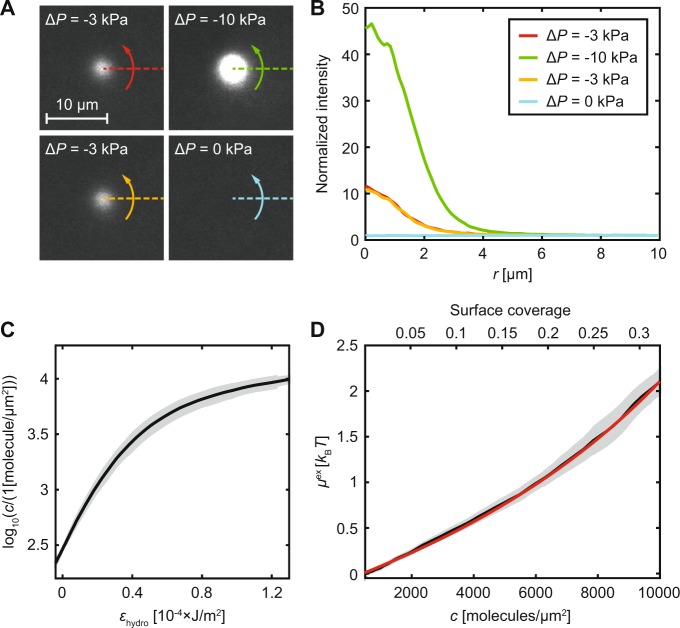
From trapping data to interaction curves. (**A**) Images of fluorescently-labelled SA being accumulated on an SLB at different applied pressures, Δ*p*. (**B**) Radially-averaged intensity profiles of the data in A. (**C**) The interaction curve of SA (mean ± SD). (**D**) The experimentally determined excess chemical potential for SA (black; mean ± SEM). The red line is the theoretical curve for a hard disk with a radius of 3.2 nm.

A description of the trapping method and how to obtain the chemical potential is described below for the protein SA. A micropipette was positioned a fixed distance above an SLB containing anchored SA, after which a negative pressure was applied through the pipette, creating an inward flow and accumulation of SA (see Fig. 2A and Supplementary Video S1). A higher pressure applied through the pipette led to greater accumulation. The relative intensity increase compared to before trapping is shown in Fig. 2B, where the intensity is radially averaged around the centre of the trap. This value can in turn be converted to a concentration of proteins at different positions in the trap (see Supplementary Methods – “*Conversion between fluorescence intensity and protein density*”). Whereas SA has been observed to crystallize at higher surface densities^12^, this was not the case in the current trapping experiments. However, it was observed that photobleaching of the trapped SA molecules, under high salt and low pH, resulted in the proteins becoming immobile (see Supplementary Fig. S1 and Supplementary Video S2). Similarly, SA also started to crystallize, without any trapping taking place, only after a region of the fluorescently-labelled proteins was photobleached for an SLB containing 10 wt% biotinylated lipids. How photobleaching triggers this apparent crystallization, which only happens at high protein concentrations, is unclear to us, but it is possible that the fluorescent groups affect the protein’s ability to crystallize.

To relate the accumulation of SA to the magnitude of the liquid flow into the pipette we note that the concentration of molecules, *c*, in the trap is at steady state related to the force on a molecule from the liquid flow, *F*_hydro_, by:^2^

1 $$\frac{\partial \mu }{\partial r}={F}_{{\rm{hydro}}}$$

where *μ* is the chemical potential of the molecule at the distance *r* from the centre of the trap. The hydrodynamic force depends linearly on the shear force *σ*_hydro_ according to^13^:

2 $${F}_{{\rm{hydro}}}={A}_{{\rm{hydro}}}{\sigma }_{{\rm{hydro}}}$$

where *A*_hydro_ is the effective hydrodynamic area of the macromolecule. The quantity *A*_hydro_ depends on the size and geometry of the studied molecule, as well as on the surface concentration, *c*, of molecules. Taller molecules generally have larger *A*_hydro_ than shorter molecules, and *A*_hydro_ decreases as *c* increases due to molecules shielding each other from the flow^13^. The shear force can in turn be determined from finite element simulations (see Supplementary Methods – “*Determining ε*_*hydro*_ *and σ*_*hydro*_ *from finite element simulations*”), and depends on the dimensions of the pipette, the applied pressure through the pipette and the distance between the pipette and the surface. Changing the applied pressure therefore enables the trapping strength to be varied and, with this, the number of accumulated molecules (see Fig. 2A,B). Combining equations (1) and (2) gives:

3 $$\mu (c)=\mu ({c}_{0})+{\int }_{0}^{{{\epsilon }}_{{\rm{hydro}}}}{A}_{{\rm{hydro}}}(c)d{{\epsilon }}_{{\rm{hydro}}}$$

where

4 $${{\epsilon }}_{{\rm{hydro}}}=-{\int }_{r}^{\infty }{\sigma }_{{\rm{hydro}}}dr$$

The product of *ε*_hydro_ and the average value of *A*_hydro_ corresponds to the energy required to bring a molecule along the membrane surface, from infinity to a distance *r* from the centre of the trap. The intermolecular interactions between hard disks of radius *a* are thermodynamically given by the chemical potential of the molecules according to:

5 $$\mu ({\Phi })={\mu }_{0}+{k}_{{\rm{B}}}T\,\mathrm{ln}({\Phi })+{k}_{{\rm{B}}}T(2{B}_{2}\Phi +\frac{3}{2}{B}_{3}{{\Phi }}^{2}+\frac{4}{3}{B}_{4}{{\Phi }}^{3}+\cdots )$$

where *Φ = c* × π*a*^2^ is the surface coverage at the molecular concentration *c*, *μ*_0_ is the chemical potential at the standard state and *B*_i_ is the i^th^ virial coefficient of the expansion^14^. For hard disks in two dimensions the first three virial coefficients take on the following values: *B*_2_ = 2, *B*_3_ = 3.128 and *B*_4_ = 4.258^14^. At low coverage, equation (5) will approach *μ*(*c*) = *μ*(*c*_0_) + *k*_B_*T* ln(*c/c*_0_), where *c*_0_ is the concentration before trapping. Inserted into equation (3), and setting *A*_hydro_ = *A*_hydro_(0), results in:

6 $$\mathrm{ln}(c)=\,\mathrm{ln}({c}_{0})+{A}_{{\rm{hydro}}}(0){{\epsilon }}_{{\rm{hydro}}}/{k}_{{\rm{B}}}T$$

A plot of ln(*c*) vs *ε*_hydro_ (henceforth called an “interaction curve”; see Fig. 2C), will therefore at low coverage have the slope *A*_hydro_(0)/*k*_B_*T*. We have previously shown that *A*_hydro_(0) can be estimated using the following empirical expression^13^:

7 $${A}_{{\rm{hydro}}}(0)=(0.65{({h}_{{\rm{c}}}/a)}^{2}+5.0({h}_{{\rm{c}}}/a)+1)\pi {a}^{2}$$

where *h*_c_ is the height and *a* the radius of the molecule. The interaction curve is a characteristic function for each studied protein, which in general is independent of how the curve is obtained (such as the magnitude of the liquid flow, the size of the pipette etc.; see also Supplementary Fig. S2).

### The interaction between globular proteins - streptavidin

The initial slope of the interaction curve for SA gave *A*_hydro_(0) = 300 ± 29 nm^2^ (mean ± SD). This value is similar to the theoretical value of *A*_hydro_(0) = 296 nm^2^, obtained using equation (7) when modelling SA as a disk with a radius of *a* = 2.8 nm and height *h*_c_ = 5 nm using the crystal structure^4^. The value of *A*_hydro_ at different concentrations will, in addition to *A*_hydro_(0), also depend on the quotient *h*_c_/*a*^13^. However, the dependence on *h*_c_/*a* is weak in the range *h*_c_/*a* = 1 to 10 (see Supplementary Fig. S3) and the function *A*_hydro_(*c*) can therefore be estimated from *A*_hydro_(0). This, together with the values from the interaction curve, can be inserted into equation (3) to give the chemical potential *μ*. Figure 2D shows SA’s excess chemical potential defined as: *μ*^ex^ = *μ* − *k*_B_*T* ln(*c*/*c*_0_). The excess chemical potential would be zero for non-interacting molecules of infinitely small size. However, *μ*^ex^ will be negative if there are attractive forces between the molecules and positive if there are repulsive forces. The finite size of the molecules will also, due to molecular overlap, give a positive contribution to the excess chemical potential. The more interactions, or the larger the molecules, the higher will the absolute value of *μ*^ex^ be. The red line in Fig. 2D is a fit of the excess chemical potential to a hard disk model (equation (5)) yielding a disk radius of 3.2 ± 0.4 nm. This is 0.4 nm larger compared to the crystal structure for SA. The increase in radius may be explained by the extra area taken up by the fluorescent Alexa Fluor® 647 groups (on average three per SA molecule), which would correspond to an effective increase in cross-sectional area of the order of 30% in total (see Supplementary Fig. S4).

### The orientation and height of CD2, CD4 and CD45 on the lipid bilayer

**Figure 3:**
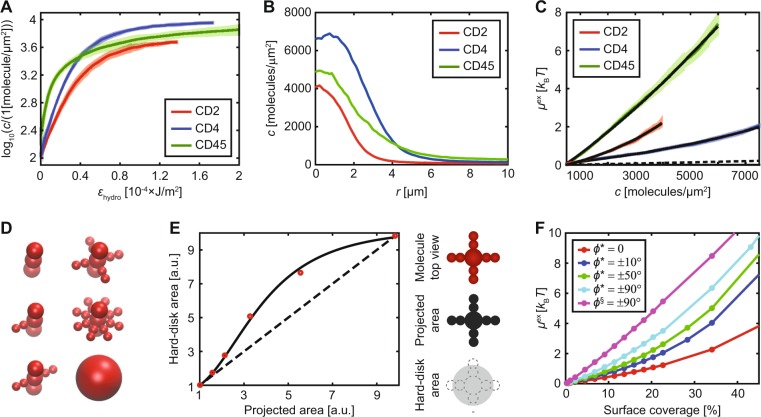
Hydrodynamic trapping of CD2, CD4 and CD45. (**A**) Interaction curves for the three proteins (mean ± SD). (**B**) Radially-averaged concentration profiles of the three proteins as a function of distance, *r*, to the centre of the trap. The maximum value of *ε*_hydro_ was 10^−4^ J/m^2^ for all three cases. (**C**) The experimentally determined excess chemical potential for the proteins (mean ± SEM). The black lines are fits to the following theoretical models: (i) “hard disks” for CD2 and CD4 and (ii) “±90° rotating rod” for CD45. The dashed line is *μ*^ex^ for a hard disk with a radius of 1.5 nm. (**D**) Different molecular constructions used in the MC simulations when investigating glycosylation. (**E**) (Left) The effective hard-disk area for a glycosylated molecule vs the projected area of the protein + sugars obtained from MC simulations. The dashed line corresponds to the hard-disk area being equal to the projected area and the solid line is a fit to equation (8). (Right) Definition of “projected area” and “hard-disk area” (shaded areas), for a molecule with the projected area equal to 3.3 a.u. (**F**) Excess chemical potential curves from MC simulations for a rod-like molecule which can rotate an angle *ϕ* around its attachment point. ^*^Molecule consists of three connected spheres. ^§^Molecule consists of six connected spheres.

We next turned to investigate the behaviour of the presumably rod-like proteins CD2, CD4 and CD45 anchored to an SLB. Both the orientation of these molecules on the SLB, as well as their effective size when including sugars, are, in contrast to SA, not well defined. Trapping experiments for each of the proteins were performed as described previously for SA. Figure 3A shows the interaction curves for the three proteins. CD2 has the shallowest slope at low surface coverage with CD4 in the middle and with CD45 having the largest slope. With a steeper slope corresponding to a higher *A*_hydro_(0) value, and thus a larger molecule, this is in agreement with CD45 being the largest followed in size by CD4 and CD2. It is of interest to compare the experimental values for *A*_hydro_(0), summarized in Table 1, with the values obtained from equation (7) using data from structural studies, assuming the proteins are “standing up”. All immune-cell proteins were approximated as a cylinder with a radius of 1.5 nm, neglecting the effect of the sugar groups on the hydrodynamic area (see Table 1 for values). The effective height of the molecules, *h*_c_, is calculated using Supplementary equation (S8) and the experimental *A*_hydro_(0) value. The results obtained are in good agreement with CD2 and CD4 standing upright on the SLB with an effective height within 7% of that obtained from the protein structures (Table 1). The slightly larger height obtained for CD2 can be attributed to the extra drag taken up by its four N-glycans, whereas this deviation would be smaller for CD4 which only has two glycosylation sites^15^. The full length of CD45 on the other hand, has previously been estimated to be approximately 40 nm^9^, which is considerably larger than the effective height of 22 nm obtained from the trapping data. The extracellular domain of CD45 consists of an N-glycosylated region (CD45d1-d4) with a height of approximately 15 nm, followed by a mucin-like region^7^. One reason for the low effective height obtained by us might be that the mucin-like region has a considerably smaller hydrodynamic area, and thus takes up less hydrodynamic force, than estimated by equation (7). Another possibility is that CD45 can be significantly tilted at the low coverage where *A*_hydro_(0) is measured. This also agrees with CD45 being more flexible than the other molecules investigated here (see below).

**Table 1:** Theoretical and experimental values of the protein radius, *a*, height, *h*_c_, and hydrodynamic area, *A*_hydro_(0).

		SA	CD2	CD4	CD45
Theory^a^	*a* [nm]	2.8	1.5	1.5	1.5
	*h*_c_ [nm]	5	7.5	11	>15
	*A*_hydro_(0) [nm^2^]	296	299	513	≥820
Experiment^b^	*A*_hydro_(0) [nm^2^]	300 ± 29	324 ± 18	505 ± 40	1536 ± 352
	*h*_c_ [nm]^c^	5.1 ± 0.4	8.0 ± 0.3	10.9 ± 0.6	22.2 ± 3.1
	*a*_hd_ [nm]^d^	3.2 ± 0.4	5.3 ± 0.2	3.7 ± 0.3	8.0 ± 0.3^e^

^a^Theory from: Darst *et al*. (SA)^4^, Davis and van der Merwe (CD2)^20^, Yin *et al*. (CD4)^8^, and Chang *et al*. (CD45)^7^.

^b^Experimental values are given as mean ± SD.

^c^Estimated height value (mean ± SD) calculated from Supplementary equations (S9) and (S10) using the experimental *A*_hydro_(0) and the theoretical *a*.

^d^Fit of *μ*^ex^ to a hard-disk model, with *a*_hd_ being the disk radius.

^e^Value at *c* = 500 molecules/μm^2^. *a*_hd_ = 5.3 ± 0.1 nm at *c* = 6000 molecules/μm^2^.

### The interaction between rod-like molecules - CD2, CD4 and CD45

Figure 3B shows the radially-averaged concentration profile for each protein when the maximum value of *ε*_hydro_ in the trap is 10^−4^ J/m^2^. Since the concentration profiles are different this also indicates that the chemical potential of the proteins will be different. This can also be observed in Fig. 3C, which shows the excess chemical potential for CD2, CD4 and CD45. The excess chemical potential for all three proteins increases considerably faster than it would if they were hard disks with a radius of 1.5 nm, and the proteins are thus significantly more repulsive. CD2 and CD4 could be modelled as hard disks with a radius of 5.3 nm and 3.7 nm, whereas CD45’s excess chemical potential curve altogether deviates from that of a hard disk. These effects can be explained by protein glycosylation and flexibility as discussed below.

### Protein glycosylation

An explanation for the larger effective radius of CD2 and CD4 is that it is due to N-glycans protruding from the protein core. The length of the sugars on CD2 and CD4 are of the order of 3 to 4 nm^15^, and can thus significantly extend the lateral dimensions of the proteins. The larger radius of CD2 compared to CD4 would then be in agreement with CD2 being more glycosylated (four glycosylation sites for CD2 compared to two for CD4)^15^. To investigate this further, MC simulations were performed for a model protein, mimicking different amounts of glycosylation (see Fig. 3D). Two parameters, the *projected area* and the *hard-disk area*, were introduced to characterize this (see Fig. 3E). The former is the total cross-sectional area of the protein and sugars, whereas the latter is the corresponding area of a hard disk with equal excess chemical potential at 1 *k*_B_*T*. The hard-disk area was found to be equal to the projected area at low sugar densities (see Fig. 3E). However, the hard-disk area can be significantly larger than the projected area at higher densities and approach the projected area of a disk with a radius equal to the protein radius + the sugar length (see Fig. 3E). The effect of glycosylation is significantly less pronounced if the protein can rotate freely around its attachment point, resulting in tilt angles between ± 90°. The hard-disk area now depends on the volume of the sugar groups instead of their projected area (see Supplementary Fig. S5). This is valid for the simplified systems modelled here where all the sugars are assumed to be in one plane of the molecule.

### Protein flexibility

Thermal motion will, if the molecule or attachment point at the surface is flexible, cause the molecule to rotate, or tilt, around its attachment point. This affects the effective width of the molecule on the surface. Figure 3F shows the results from different MC simulations where a rod-like molecule can rotate around its attachment point. For a rod with a 3:1 height-to-width ratio, the effective width of the protein increases by 1.4, 1.7 and 2.3 times that of a rigid rod (at 1 *k*_B_*T*), when the protein can rotate ±10°, ±50° and ±90° around its attachment point, respectively. The effective width increment also depends on the rod length, with longer rods showing a larger increase (see Supplementary Fig. S6). The excess chemical potential curves for the latter differ from the hard-disk curves, adopting a near linear dependence on surface coverage (see Fig. 3F), indicating that the repulsive interactions increase constantly with surface coverage.

### The interaction between rod-like molecules - CD2, CD4 and CD45 (continued)

If CD2 and CD4 can rotate ±10° around the vertical axis as previously suggested by Polley *et al*.^16^, and the effective area per sugar group is 4 nm^2 ^^15^, this yields an effective hard-disk radius of 3.6 nm for CD4 and 4.9 nm for CD2 (1.4 × the hard disk radius from Fig. 3E). For this it is assumed that the sugars on CD4 and CD2 are similar in size and that CD4 has two sugars, whereas CD2 has four^15^. These values are in qualitative agreement with the experimental observations, illustrating how glycosylation can significantly increase the intermolecular repulsion between proteins.

The excess chemical potential for CD45 is considerably higher than for the other proteins, indicating that CD45 is more repulsive than CD2 and CD4. However, compared to a hard-disk model the relative repulsion (effective cross-sectional area) decreases at higher concentrations. This agrees with a model wherein CD45 can freely rotate around its attachment point at the surface (see Fig. 3F). The experimental data were fit to MC simulated curves of freely rotating rods of varying height-to-width ratio (see Supplementary Fig. S6). The fit for a 40 nm long molecule gave a radius of 3.8 nm, similar to the effective radius of CD4. The effective cross-sectional area for CD45 at low coverage (500 molecules/μm^2^) corresponds to 200 nm^2^ but is less than half of this value (88 nm^2^) at 6000 molecules/μm^2^. In fact, the effective cross-sectional area at 6000 molecules/μm^2^ is similar to the cross-sectional area of CD2.

### Trapping two types of proteins

**Figure 4:**
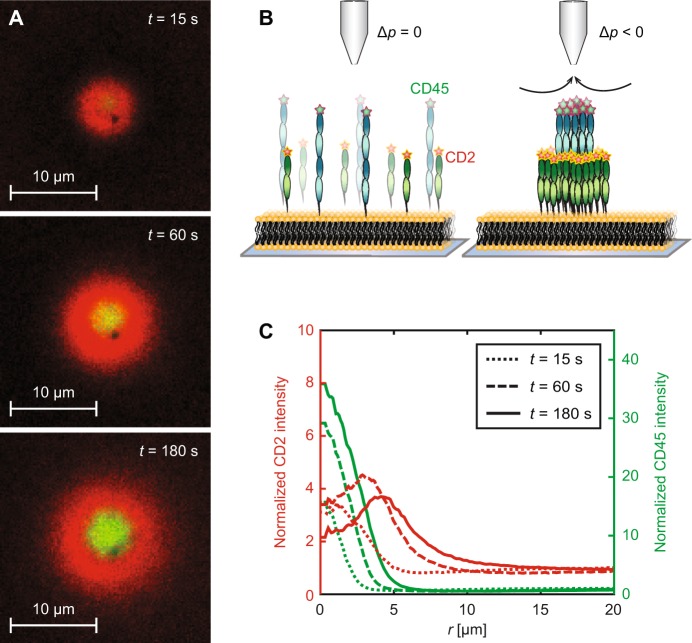
Simultaneous trapping of CD2 and CD45. (**A**) Trapping of CD2 (red) and CD45 (green) after pressure is applied at *t* = 0. (**B**) Scheme showing the double trapping. (**C**) Radial line profiles of CD2 (red lines) and CD45 (green lines) at different times.

Experiments were also performed for SLBs containing CD45 together with the protein CD2 to investigate the distribution of two differently-sized proteins in the trap (see Supplementary Video S3). Figure 4 shows the distribution of both CD2 (red) and CD45 (green) at various times after the trap is turned on. Both proteins initially accumulate in the centre of the trap, however, CD2 is partially excluded from the centre as the concentration of CD45 increases. The observed ring-like distribution of CD2 around CD45 can be explained by a combination of (i) shielding of CD2 by the larger CD45 molecules and (ii) competition for the free area in the centre of the trap (see Supplementary Methods - “*Trapping of two proteins*” and Supplementary Fig. S7). The accumulation of CD45 also weakens (see Supplementary Fig. S8) with an *A*_hydro_(0) value of approximately 1000 molecules/μm^2^, compared to 1500 molecules/μm^2^ without CD2. This agrees with CD2 partially shielding CD45 from the flow. Similar, but less pronounced, data were obtained when investigating the trapping of CD4 together with CD45 (see Supplementary Fig. S9).

## Discussion

Hydrodynamic trapping makes it possible to investigate how membrane-anchored molecules interact over a wide range of concentrations, approaching the concentrations of densely packed proteins. High surface coverages have previously been difficult to study since they have required unpractically high concentrations of protein in solution when anchoring the molecules to the lipid bilayer. In this study, the amounts of immune-cell proteins used for each experiment were 10 to 100 ng, and the surface coverage in the centre of the trap exceeded 30%. Higher surface coverage can be obtained by increasing the applied pressure over the pipette which, in the current experiments, was of the order of 10 kPa. Doubling the applied pressure for the SA experiments presented in Fig. 2 would, for example, increase the maximum surface coverage in the trap from 32% to 48%, assuming that the proteins still behaved as hard disks with a radius of 3.2 nm. It is at these high coverages possible to investigate the aggregation of protein molecules and what types of conditions favour this. For the investigated proteins it was only SA that could be induced to aggregate, and this in turn required the use of high salt and low pH (650 mM NaCl and a pH of 3.6) and for the proteins to be photobleached in the trap (see Supplementary Fig. S1). The molecular reason for this behaviour remains unknown but demonstrates how the hydrodynamic trap can induce protein clustering under suitable conditions. None of the other proteins exhibited tendency to dimerize. There is conflicting data on whether the studied T-cell proteins dimerize on the cell surface or not^17–19^, however, our results indicate that, if this is the case, it is not driven by interactions between the extracellular parts of the proteins.

It has also been observed that deglycosylation of these molecules aids in forming protein crystals^7,20^, thus indicating that the N-linked glycans protruding from the protein core increases the intermolecular repulsion. This was also confirmed by our experiments, which combined with MC simulations, allowed us to investigate how different degrees of glycosylation influences the interaction. It was found that even sugars constituting a minor part of the total protein mass can have a dominating effect on the intermolecular interaction. This effect is especially true for rigid molecules whose orientation relative to the lipid bilayer remains essentially constant. The effective repulsion of the glycosylated protein can here be calculated from the projected area of the sugar groups and will have a maximum relative effect for intermediate levels of glycosylation (see Fig. 3E). The effect of glycosylation will be less for flexible or freely rotating proteins, and in the latter case the increase in repulsion depends on the added total volume of the sugars. The difference comes from having all sugars in the same plane(s) with no or little rotation, but in different, non-coincident, planes when the molecule can rotate ±90° around its attachment point. Thus, glycosylation is expected to have the largest relative effect for rigid proteins. The extra repulsion that glycosylation gives rise to for CD2 and CD4 makes them less likely to dimerize or aggregate on the cell surface, which might be important in preventing inappropriate signalling. It can also reduce the effective affinity of these proteins for ligands on a contacting cell, since ligand binding would lead to an accumulation of proteins in the cell-cell contact which would be counteracted by the repulsion.

Protein rotation, or flexibility, around the anchoring point at the surface can also, on its own, significantly affect the chemical potential and how it varies with protein coverage. The larger the rotation, the higher is the chemical potential, as shown in Fig. 3F. This effect increases with the length of the studied protein. The reason for the extra repulsion is that the rotation causes the protein to occupy a larger area on the lipid bilayer compared to when the protein is standing upright. This effect increases with the length of the protein and with the maximum rotation angle. When the concentration of proteins gets higher, the initially non-constrained angles decrease, resulting in the protein standing upright due to steric hindrance (maximum entropy). Each protein is now taking up less area on the surface, however, constraining the rotation angles will have a cost in free energy. Overall, this results in an excess chemical potential with a more linear relationship to protein coverage compared to that for a non-rotating hard disk, as indeed was measured for CD45.

Both the orientation and the effective height of CD45 on the cell surface are presently unknown^7^, but have important consequences since the size-induced exclusion of these molecules from the contact between immune cells is the key mechanism in the kinetic-segregation model explaining T-cell triggering^11^. Both the lower value of *A*_hydro_(0) compared to the predicted value for an upright molecule using equation (7), as well as the linear shape of the excess chemical potential vs protein coverage, would be in agreement with a model where CD45, or its mucin-like region, is free to rotate around the attachment point, at least at low protein coverage. If the protein dimerized or aggregated, this would limit the possible angles that CD45 could rotate through, thus increasing the free energy of the system. Protein flexibility will thus counteract aggregation in this case. Flexibility will also lower the effective height of the protein, which will make the protein less excluded from narrow cell-cell contacts compared to when the protein is standing vertically all the time^21,22^. However, when the protein coverage increases this will force CD45 to adopt a more upright position. The high density of CD45 and CD43 on the T-cell surface, approximately 1000 molecules/μm^2^ for each protein^7,23^, would result in the effective cross-sectional area of CD45 being approximately 40% lower than at infinite dilution. This implies that there would be a reduction in allowed tilt angles and a more upright position of CD45 on the T-cell surface compared to an SLB at low coverage. Another factor that could influence the chemical potential is molecular reorganization of the mucin-like region of CD45 from a brush-like to a more extended form. However, this region is assumed to be rather rigid^24^, which would reduce the influence of this effect. It should also be stressed that it is the chemical potential that is measured, and different phenomena can give rise to similar chemical potentials. Depending on the properties, it might be necessary to vary other parameters in the system, such as pH, salt concentration and amount of glycosylation, and combine this with modelling or simulations, in order to be able to determine the effect of different parameters on the molecular interactions.

Only the extracellular domain of the T-cell proteins was studied in this work. These proteins were anchored to the SLB via a polyhistidine tag at the position where the transmembrane domain starts, thus mimicking the position of the extracellular domain *in vivo*. However, it is possible that the transmembrane domain, as well as the composition of the membrane, can affect the behaviour and orientation of the protein on the membrane. Complementary measurements of, for example, the effective height of the protein on live cells could aid in better understanding this^25,26^. Another possibility is to do hydrodynamic trapping on the full protein, incorporated in, for example, cushioned SLBs^27^. However, the relative differences in orientation between the proteins investigated in this study, and the effects of glycosylation and molecular flexibility, are not expected to be significantly altered by omitting the transmembrane domain.

We finally investigated how having more than one type of molecule on the surface affects the distribution in the trap. It was observed that the concentration of the largest molecule was similar to that when trapping the protein alone, while the smaller molecule became segregated from the centre of the trap (see Fig. 4). It is thus possible to locally segregate molecules based on size, with the largest molecules being in the centre of the trap. It would be feasible to increase the size of a protein by binding an antibody to the molecule, coupled to a bead to increase its size further. This could make it possible to locally increase the concentration of a certain protein even in a more complex setting, such as a live cell surface. Hydrodynamic trapping is thus a versatile method that can be used to better understand how membrane-associated molecules interact. The method could also be extended to the study of a range of other interactions, including, for example, dimer formation and aggregation of membrane-bound proteins.

## Methods

### Lipid and vesicle preparation

Vesicle solutions containing (i) 0.05% of 1,2-dipalmitoyl-sn-glycero-3-phosphoethanolamine-N-(cap biotinyl) (sodium salt) (biotin-PE, Avanti® Polar Lipids, Inc) mixed with 99.95% of 1-palmitoyl-2-oleoyl-sn-glycero-3-phosphocholine (POPC, Avanti® Polar Lipids, Inc) or (ii) 5% or 10% 1,2-dioleoyl-sn-glycero-3-[(N-(5-amino-1-carboxypentyl)iminodiacetic acid)succinyl] (nickel salt) (DGS-NTA, Avanti® Polar Lipids, Inc) mixed with 95% or 90% POPC, respectively, were prepared at a concentration of 0.5 mg/mL by the following protocol. The different lipids were first mixed in 100 µL chloroform. To remove the chloroform, the lipids were dried using a N_2_ gas flow for 10 minutes. After evaporation of chloroform the vesicles were suspended and thoroughly mixed in 1 mL of filtered (0.2 µm Minisart® Syringe filter, Sartorius) washing buffer: 150 mM NaCl, 10 mM 2-[4-(2-hydroxyethyl)piperazin-1-yl]ethanesulfonic acid (HEPES, Sigma), pH 7.4 for all lipids. The vesicles were then incubated on ice for 1–2 h followed by tip sonification with a CV18 model tip sonicator (Chemical instruments AB) for 15 minutes with a pulse time of 10 s and an amplitude of 55%. The lipid stock solutions were stored at −20 °C in chloroform and the vesicle solutions at 4 °C until used.

### Cover slide preparation and protein loading

0.15 mm thick, round glass slides (number one coverslips Ø 25 mm, Thermo Fisher Scientific) were cleaned for 30 min in 80 °C heated piranha solution (mixture of 75% sulfuric acid (99.9%, Sigma) and 25% hydrogen peroxide (30%, Sigma)). After the piranha wash the glass slides were rinsed for one minute on each side with deionized water and then dried with N_2_ gas. A silicon well (Silicon isolators, 12 × 4.5 mm diameter, 1.7 mm depth; Grace Biolabs) was cleaned with ethanol, paper dried and dust particles were removed with tape. The clean glass slide was pressed on the silicon well and placed in an Attofluor® Cell Chamber (Thermo Fisher Scientific). The well was filled with washing buffer and left for sample preparation covered with a Petri dish to avoid dust in the buffer. Vesicles were diluted 1:10 in 30 µL of the washing buffer, added to the prepared well and incubated for one hour at room temperature. This allowed for formation of a fluid and continuous SLB on the glass surface. Non-ruptured vesicles were after the incubation washed away and the proteins were added to the well. 40 µL of Streptavidin, Alexa Fluor® 647 conjugate (Thermo Fisher Scientific) were added at a concentration of 20 ng/µL and bound to the biotin-PE lipid bilayers.

Binding of proteins to the DGS-NTA lipids required a histidine tag covalently bound to the proteins. Thus, recombinant rat CD2 with a modified C-terminus containing a double histidine tag, as well as human CD45RABC and human CD4 containing one histidine tag, were made to bind to the DGS-NTA lipids. The proteins were fluorescently labelled with the dyes Alexa Fluor® 488 and Alexa Fluor® 647 using an Alexa Fluor antibody labelling kit from Thermo Fisher Scientific. All proteins were diluted in 150 mM NaCl and 10 mM HEPES, pH 7.4. Whereas 30 µL of 0.5 ng/µL CD2 or CD4 were loaded on the DGS-NTA lipid bilayer, 30 µL of 3 ng/µL of CD45 were used. The proteins were allowed to settle for one hour and excess proteins were washed away with washing buffer.

### Microscopy setup

The fluorescently-labelled molecules were studied with a Nikon Apo TIRF 60× magnification oil immersion objective on a Nikon Eclipse TE2000-U microscope equipped with a Hamamatsu ORCA-Flash4.0 LT Digital CMOS camera (C1140-42U). The sample was illuminated using Cobolt MLD compact diode lasers operating at a wavelength of 488 nm (20 mW) and 638 nm (140 mW). The acquired images were 512 pixels by 512 pixels with a pixel size of 0.22 µm by 0.22 µm.

For fluorescence recovery after photobleaching (FRAP) analysis a series of pre-bleached images was taken under the above stated settings. The field diaphragm was closed to a small circular area and left for six seconds to illuminate and bleach a small region on the visible SLB. In this time the ND filter (ND 2.0) was removed for three seconds from the beam path resulting in efficient bleaching of the illuminated area. The ND filter and field diaphragm were returned to their original positions and the recovery was followed for 106 seconds. For all experiments the time between frames was two seconds.

### Pipette pulling and characterization

**Figure 5:**
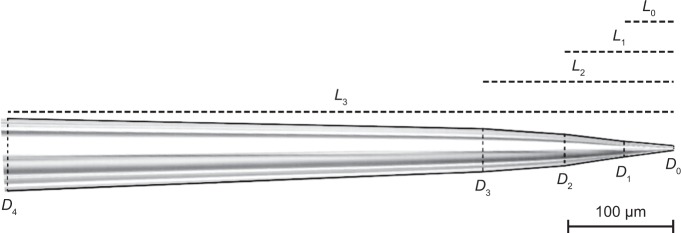
Image of a micropipette captured with a 20× magnification air objective. Solid lines denote the outer walls of the pipette, dashed lines denote the start of a new conical segment and the dotted lines represent the distance between the pipette tip and the conical segments.

Micropipettes with a size of 2–5 µm in inner tip diameter were pulled with a flaming/brown type micropipette puller (model P-97; Sutter Instruments) from borosilicate glass capillaries with an inner diameter of 0.58 mm and an outer diameter of 1.0 mm. An image of the pipette was acquired in bright field mode with a 20× magnification air objective (1.54 pixels/µm). The outline of the pipette was manually determined and can be described as multiple connected conical segments (see Fig. 5). The dimensions of the segments were used to determine the flow profile outside the pipette using finite element simulations (see Supplementary Methods - “*Simulations of the flow and ion current in the pipette*”).

### Protein accumulation under hydrodynamic forces

The pulled micropipette was mounted on a custom-made micropipette holder for the hydrodynamic trapping experiments. The holder can be moved in the *x*-, *y*- and *z*-direction with nm-precision using a 3D piezoelectric positioning system (P-611.3 s NanoCube®XYZ-system, Physik Instrumente). Manual stages were used for more coarse positioning in the *x*- and *y*-direction and for coarse motion in *z*-direction a motorized stage from Thorlabs (T-cube Brushless DC Servo controller, PT1/M) were used. This assures that the mounted pipette can be centred over the sample. In addition, an Ag/AgCl electrode was inserted in the pipette. To measure the distance of the pipette to the SLB the ion current between the electrode in the pipette and an Ag/AgCl reference electrode in the bath solution around the pipette was measured. The electrode in the micropipette was chlorinated before each experiment using bleach to guarantee a stable ion current between the two electrodes. When the pipette approaches the surface, the resistance between the two electrodes increases. This increase, around 0.5–1% in the current experiments, can be converted to an actual distance of the pipette to the surface when the dimensions of the pipette are known^2^. The pipette was then moved away between 2 µm to 6 µm to create a bigger area for the trapping region.

Accumulation of proteins was initiated by applying negative pressure over the micropipette. The proteins were accumulated under different pressures: −3.0 ± 0.3 kPa, −9.7 ± 0.3 kPa and −19.4 ± 0.3 kPa. Between each pressure change, and measurement, the concentration of the proteins in the trap had reached equilibrium. Finally, the proteins were released by turning off the pressure. The accumulation was captured with the microscope settings stated above, but with a time frame of 15 seconds between each image.

### Analysis of the trapping data

The acquired fluorescence images were analysed using a custom-written MATLAB® (MathWorks Inc.) program to give an interaction curve for each trapping event. A fluorescence image of the trapped region was acquired after the concentration of proteins had reached steady state and the intensity was radially averaged around the centre of the trapped region. The radial fluorescence intensity was next converted to a molecular density by a conversion factor obtained from single molecule imaging as described in Supplementary Methods - “*Conversion between fluorescence intensity and protein density*”. Corresponding values for *ε*_hydro_ at different radial positions were determined as described in Supplementary Methods - “*Determining ε*_*hydro*_ *and σ*_*hydro*_ *from finite element simulations*”. Plotting the molecular concentration *c* as a function of *ε*_hydro_ finally gave the interaction curve.

### Metropolis-Hastings Monte Carlo simulations

All MC simulations were performed using the Faunus software package^28^. Molecules were treated as a sequence of connected spheres, of which one is grafted to a hard, planar surface. Molecules were allowed to rotate and move along the surface during each MC step, and periodic boundary conditions were applied in the *x*- and *y*-direction. A purely repulsive, truncated and shifted Lennard-Jones pair potential was used for inter-sphere interactions^29^. This resulted in a slightly larger disk (7.5% larger radius) than when using a hard disk potential, which was corrected for by multiplying all surface coverages with 1.075 × 1.075. Electrostatic interactions were neglected due to the high salt concentration used and small surface charge density of the proteins.

In order to constrain rotation, the sphere furthest from the surface may be exposed to a quadratic potential: *u* = *k*_*f*_ (*z-L*_*max*_)^2^, where *z* is the distance from the surface, *L*_*max*_ is the length when standing perpendicular to the surface, and *k*_*f*_ is a spring constant. Thus, for the limit *k*_*f*_ → 0 the molecules are freely rotating, while if *k*_*f*_ → ∞ they stand perpendicular to the surface. We investigated both soft (*k*_*f*_ = 1 *k*_*B*_*T*/Å^2^), stiff (*k*_*f*_ = 5 *k*_*B*_*T*/Å^2^) and very stiff (*k*_*f*_ = 50 *k*_*B*_*T*/Å^2^) springs, giving rise to 95% probabilities of ±50°, ±10° and 0° degrees deviations from the surface normal, respectively. MC simulations with 1 million steps were run with different types of studied molecules, investigating the effect of length and topology of the molecule on the chemical potential. The excess chemical potential as a function of surface coverage was calculated by the Widom insertion method^29^. Initially, all spheres of the molecule had a diameter of 0.1 nm and the box size was 2 × 2 × 2 nm^3^. A bigger simulation box was used (4 × 4 × 4 nm^3^ or 6 × 6 × 6 nm^3^) when modelling the effect of glycosylation. The protein part of the molecule was here substituted by three connected spheres with a diameter of 0.3 nm while the sugars were approximated with two connected spheres with a diameter of 0.16 nm (see Fig. 3D). The topology of the molecules was fixed during the simulation and no additional internal flexibility was allowed. The surface coverage, where each of the excess chemical potential curves was 1 *k*_B_*T*, was interpolated from the simulated curves in Supplementary Fig. S10 and the ± 10° data was used to produce Fig. 3E. The data was fit to the following empirical formula:

8 $${A}_{\mathrm{hard}\mathrm{disk}}=\frac{{p}_{1}{({A}_{{\rm{projected}}}-1)}^{2}+{p}_{2}({A}_{{\rm{projected}}}-1)+1}{{p}_{1}{({A}_{{\rm{projected}}}-1)}^{2}/9.82+1}$$

where *A*_hard disk_ and *A*_projected_ is the *hard-disk area* and *projected area*, respectively, normalized to the projected area without sugars. Equation (8) levels of at 9.82, which corresponds to the (normalized) projected area of a sphere with the radius given by the protein radius + sugar length in our simulations (see Figs 3D,E), and increases approximately linearly with *A*_projected_ at low levels of glycosylation. The coefficients *p*_1_ = 0.66 and *p*_2_ = 0.95 were determined by fitting equation (8) to the experimental data.

## Electronic supplementary material

Supplementary Information

Supplementary Video S1

Supplementary Video S2

Supplementary Video S3
