# Supplementary material for: Hydrodynamic trapping measures the interaction between membrane-associated molecules
Source: Sci Rep. 2018 Aug 20;8:12479. doi: 10.1038/s41598-018-30285-0 (PMC6102267; doi:10.1038/s41598-018-30285-0)
Supplement: Supplementary file 1 — Supplementary Information [file 41598_2018_30285_MOESM1_ESM.pdf]

SUPPLEMENTARY INFORMATION

Hydrodynamic trapping measures the interaction between membrane-associated molecules

Victoria Junghans[§], Jana Hladilkova[§], Ana Mafalda Santos[‡], Mikael Lund[§], Simon J. Davis[‡], and Peter Jönsson[§]*

[§] Department of Chemistry, Lund University, SE-22100 Lund, Sweden.

[‡] Weatherall Institute of Molecular Medicine, University of Oxford, Oxford OX3 9DS, UK.

*Correspondence: peter.jonsson@fkem1.lu.se

Supplementary Figures

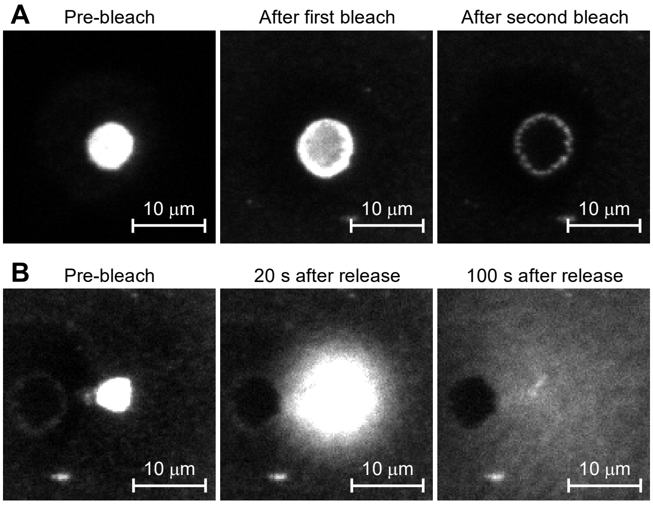

Supplementary Figure S1. A) Photobleaching trapped SA results in a region of immobile molecules at the center of the trap. **B)** Trapping without photobleaching in an area next to the images in A. The SA diffuses away when the trap is turned off. The experiments were performed on an SLB consisting of 1:10 biotin-PE:POPC and half of the SA was labelled with Alexa Fluor® 488 and the other half with Alexa Fluor® 647. The images are from the 488 channel, but a similar behavior was observed in the 647 channel. The buffer solution used contained 10 mM HEPES, 650 mM NaCl and had a pH of 3.6.

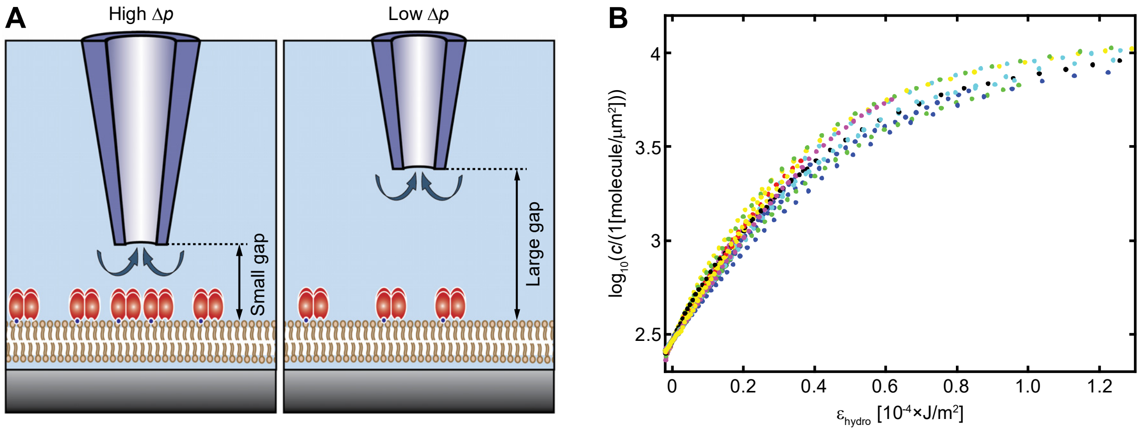

Supplementary Figure S2. A) Trapping with high pressure at a small distance to the surface gives a high trapping force, whereas trapping with low applied pressure and/or a large gap between the pipette and the surface gives a low trapping force. **B)** Interaction curves from 19 different trappings of SA, taken with both low and high trapping force as seen in the different maximum ε_{hydro} values. The shape of the interaction curves is essentially independent of the trapping strength.

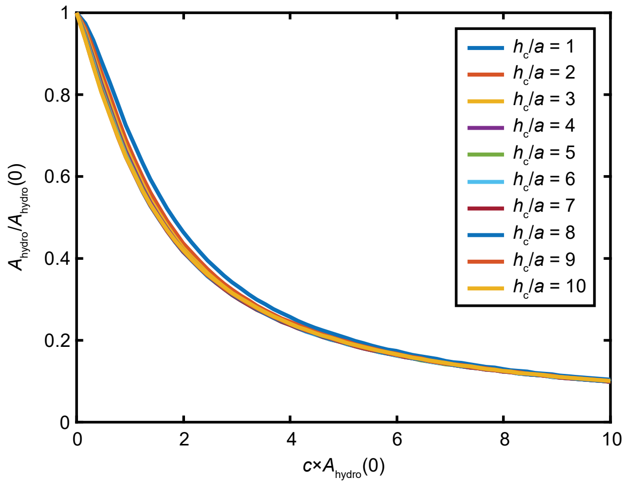

Supplementary Figure S3. Calculated values of A_{hydro} as a function of $c \times A_{hydro}(0)$ for different ratios of h_c/a using equation (14) in Jönsson and Jönsson.[1] The value of A_{hydro} is only weakly depending on h_c/a for a fix value of $A_{hydro}(0)$.

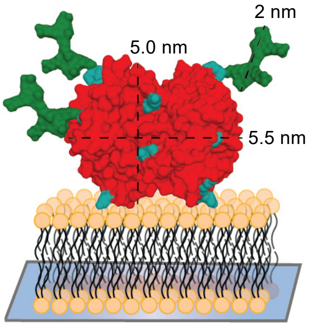

Supplementary Figure S4. Schematic presentation of labelled SA with three Alexa Fluor 647. The attached labels effectively increase the cross-sectional area of SA. Red: SA; Cyan: Lysines (possible labelling positions); Green: Alexa Fluor 647.

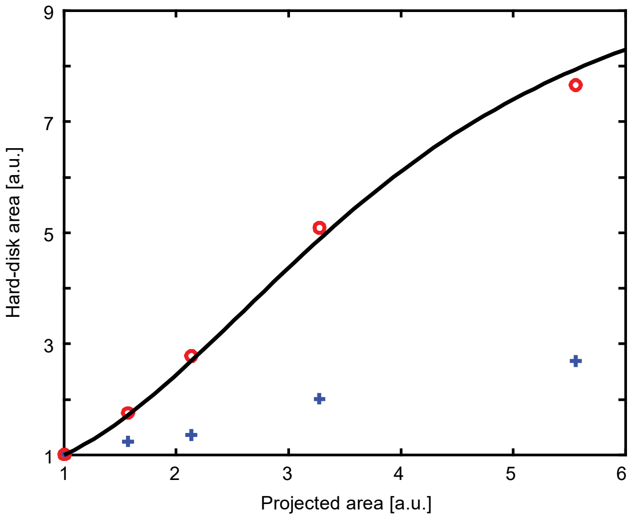

Supplementary Figure S5. Simulated effective hard-disk areas for a model protein (at 1 k_BT) with varying amount of glycosylation as a function of the projected area. The molecular complex is allowed to rotate ±10° (o) and ±90° (+) around the attachment point at the surface and the data is normalized to the value without sugars (projected area equals one). The solid line is a fit of the ±10° data to equation (8).

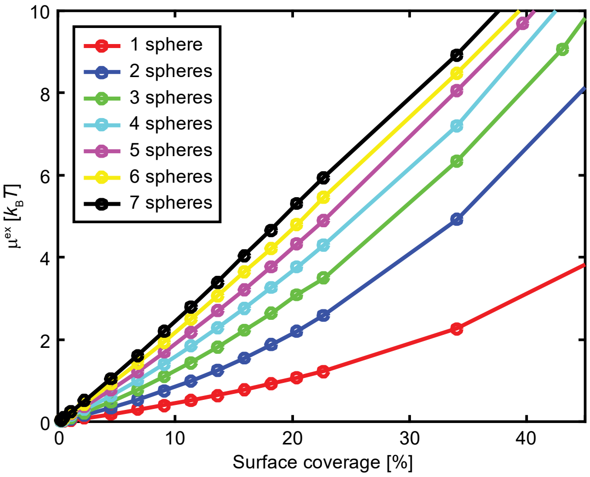

Supplementary Figure S6. MC simulated excess chemical potentials as a function of surface coverage for molecules constructed from linearly-connected spheres (1 = sphere, 7 = rod-like). The formed molecules can freely rotate around the attachment point at the surface.

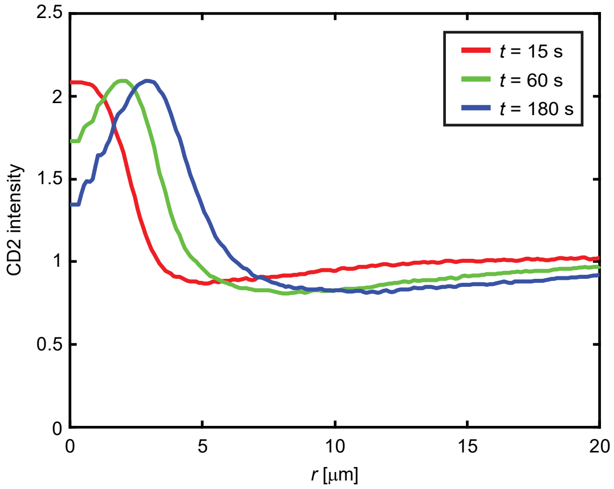

Supplementary Figure S7. Theoretically calculated radial distribution functions of the CD2 distribution in the CD2-CD45 trap at the times indicated in Fig. 4.

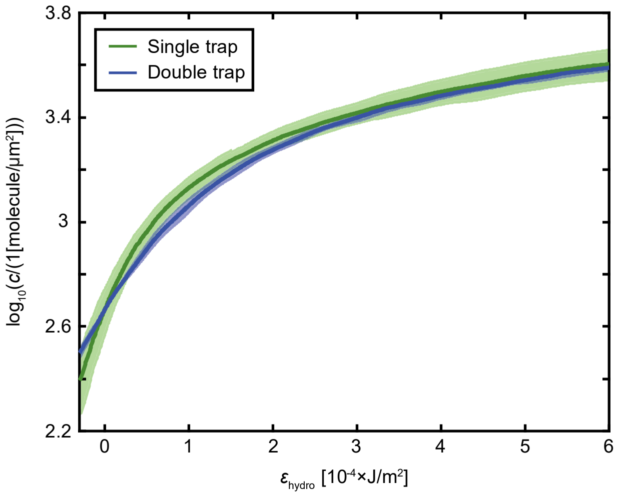

Supplementary Figure S8. Interaction curves for CD45 when trapped alone (green curves; n = 19) and when trapped in the presence of CD2 (blue curves; n = 3). The data is presented as mean ± SD.

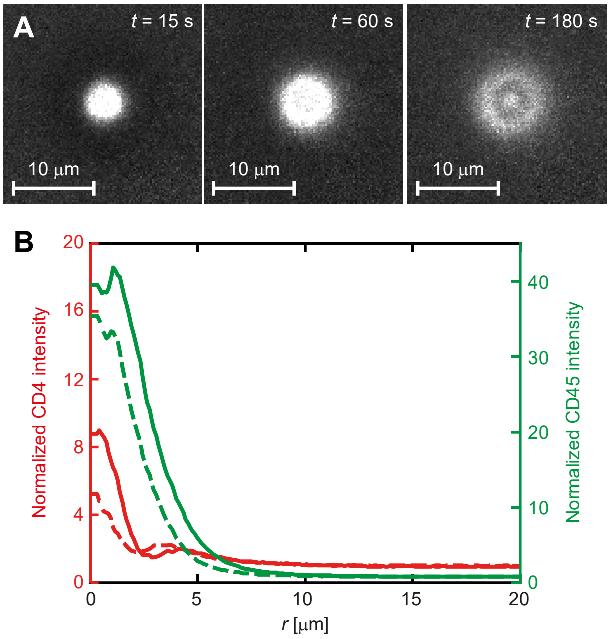

Supplementary Figure S9. A) Accumulation of CD4 at different times in a trap containing both CD4 and CD45. Trapping starts at t = 0. **B)** Steady state, radial line profiles of CD4 (red lines) and CD45 (green lines) at two different applied pressures (dashed lines: Δp = -10 kPa, solid lines: Δp = -20 kPa).

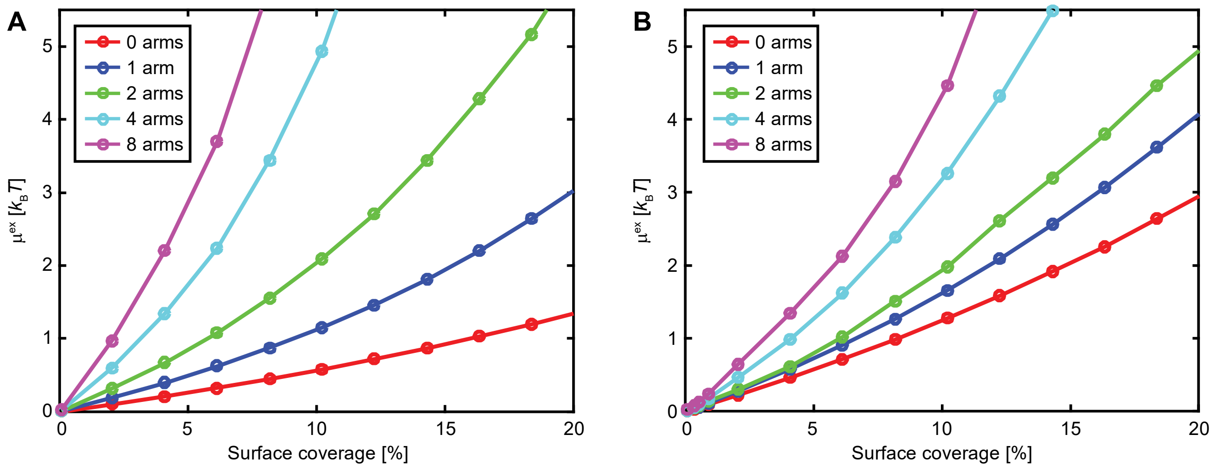

Supplementary Figure S10. The results from MC simulations where different amounts of connected spheres are forming a glycosylated protein, as shown in Fig. 3D. The formed structure can rotate **A)** ±10° and **B)** ±90° around the attachment point at the surface.

Supplementary Methods

Conversion between fluorescence intensity and protein density

A 1 pM solution of labelled proteins was added to a silicone well on a glass cover slide. The proteins were allowed to bind for 10 minutes after which the protein solution was replaced with buffer solution. This resulted in 100-200 protein molecules binding randomly in the microscope field of view, where each molecule could be individually distinguished. At least three different areas were analysed for each protein. The molecules were detected and counted using an implementation of the particle detection algorithm by Crocker and Grier.[2] The sum of the intensity from each molecule, within a radius of 4 pixels from the centre of each detected molecule, was determined and subtracted with the average intensity outside the molecules to correct for non-zero background. The fluorescence intensity per pixel, I, was converted to a protein density, c, using:

$$c = I/\left(A_{\mathrm{pixel}} I_{\mathrm{single}}\right) \tag{S1}$$

where A_{pixel} is the area of a pixel in the image and I_{single} is the average intensity from a protein molecule under the same illumination and microscope settings as used in the trapping experiments. The standard deviation of the obtained I_{single} values, from the different experiments, was 5% or smaller. The obtained conversion factors were finally corrected for all protein molecules not being labelled. This was mainly necessary for CD2 where the average number of dyes per molecule was 0.75. In short, the step change in intensity of the protein when subjected to photobleaching, I_{step}, was determined which corresponds to the intensity from one dye. The mean number of dyes per protein was determined using a NanoDrop spectrophotometer (Thermo Fisher Scientific). Multiplying I_{step} with the average number of dye molecules per protein gave the corrected value of I_{single} for CD2.

Determining $\varepsilon_{\mathrm{hydro}}$ and σ_{hydro} from finite element simulations

Different factors such as the geometry of the pipette, the distance between the pipette tip and the SLB and the pressure applied over the pipette affects $\varepsilon_{\mathrm{hydro}}$. The distance between the tip of the pipette and the underlying SLB can be related to the change in ion current as the pipette approaches the surface using finite element simulations.[3] To avoid having to do a new simulation for each experiment, the following approximate expression, valid for the low-tapered pipettes used in this work, was used:

$$R_{\mathrm{pipette}} = \frac{1}{K\pi R_0}\left(\frac{1}{\tan(\phi_{\mathrm{eff}})} + k(h/R_0)\right) \tag{S2}$$

where R_{pipette} is the electric resistance over the pipette, K is the ion conductivity in the medium, R_0 the inner tip radius of the pipette and $k(h/R_0)$ a function of the dimensionless parameter h/R_0 with h being the distance between the tip of the pipette and the SLB. The inner tip radius is obtained from D_0 in Fig. 5 by multiplying with 0.29, where it is assumed that the ratio between inner and outer diameter of the pipette is constant along the length of the pipette ($R_{\text{inner}}/R_{\text{outer}}$ = 0.58). The parameter ϕ_{eff} is the voltage inner half cone angle of the multi-segmented pipette, and is defined by:

$$\frac{1}{\tan(\phi_{\text{eff}})} = \frac{1}{\tan(\theta_0)} + \frac{D_0}{D_1}\left(\frac{1}{\tan(\theta_1)} - \frac{1}{\tan(\theta_0)}\right) + \frac{D_1}{D_2}\left(\frac{1}{\tan(\theta_2)} - \frac{1}{\tan(\theta_1)}\right) + \cdots \quad \text{(S3)}$$

where:

$$\tan(\theta_{\text{i}}) = 0.29 D_{\text{i}}/L_{\text{i}} \quad \text{(S4)}$$

and it is assumed that the ratio between inner and outer diameter of the pipette is constant along the length of the pipette. It is enough to do a series of finite element simulations at different heights h above a surface for a reference pipette after which Supplementary equation (S2) can be used to determine R_{pipette} and its dependence of h for an arbitrarily-sized pipette.

The value for $\varepsilon_{\text{hydro}}$, or σ_{hydro}, can also be determined from finite element simulations.[3] The shear force σ_{hydro} will, in the creeping flow regime, be given by:

$$\sigma_{\text{hydro}}(r) = \frac{\eta Q}{R_0^3} j(r/R_0, h/R_0) \quad \text{(S5)}$$

where η is the viscosity of the fluid and j is a function of the two parameters r/R_0 and h/R_0, where r is the radial distance from the centre of the trap at the surface and h is again the distance between the pipette tip and the surface. The parameter Q is the liquid flow rate out of the pipette, which for the small cone angles considered in this work can be shown to have the following approximate dependence on the applied pressure Δp over the pipette:

$$Q = \frac{3\pi R_0^3 \Delta p}{8\eta}\left(\frac{1}{\tan(\theta_{\text{eff}})} + f(h/R_0)\right)^{-1} \quad \text{(S6)}$$

where f is a function that only depends on the parameter h/R_0 and θ_{eff} is the pressure inner half cone angle which for a pipette with multiple segments is given by the expression:

$$\frac{1}{\tan(\theta_{\text{eff}})} = \frac{1}{\tan(\theta_0)} + \frac{D_0^3}{D_1^3}\left(\frac{1}{\tan(\theta_1)} - \frac{1}{\tan(\theta_0)}\right) + \frac{D_1^3}{D_2^3}\left(\frac{1}{\tan(\theta_2)} - \frac{1}{\tan(\theta_1)}\right) + \cdots \quad \text{(S7)}$$

The value for σ_{hydro} as a function of r was determined for a reference pipette at a series of different values of h using finite element simulations as previously described.[3] With this tabulated data for the reference pipette and Supplementary equations (S5) and (S6) it is possible to determine σ_{hydro} for an arbitrarily-sized pipette at different distances h from the surface.

Converting from A_{hydro} to h_c

Equation (5) can be solved in terms of h_c/a for a specific value of $A_{\text{hydro}}(0)/\pi a^2$ resulting in:

$$h_c/a = -\frac{2.5}{0.65} + \sqrt{\left(\frac{2.5}{0.65}\right)^2 - \frac{1}{0.65} + \frac{A_{\text{hydro}}(0)}{0.65\pi a^2}} \quad \text{(S8)}$$

The mean value (< >) and standard deviation (SD) of this ratio is:

$$\langle h_c/a \rangle = -\frac{2.5}{0.65} + \sqrt{\left(\frac{2.5}{0.65}\right)^2 - \frac{1}{0.65} + \frac{\langle A_{\text{hydro}}(0)\rangle}{0.65\pi a^2}} \quad \text{(S9)}$$

and

$$\text{SD}(h_c/a) = \frac{1}{2\sqrt{\left(\frac{2.5}{0.65}\right)^2 - \frac{1}{0.65} + \frac{\langle A_{\text{hydro}}(0)\rangle}{0.65\pi a^2}}} \frac{\text{SD}\left(A_{\text{hydro}}(0)\right)}{0.65\pi a^2} \quad \text{(S10)}$$

Trapping of two proteins

For the trapping of CD2 and CD45, it is as a first approximation assumed that the chemical potential of CD45 is given by:

$$\mu_{\text{CD45}} = k_B T \ln\left(c_{\text{CD45}}/c_{\text{CD45,0}}\right) + \mu^{\text{ex}}_{\text{CD45}}(c_{\text{CD45}}) - \mu^{\text{ex}}_{\text{CD45}}\left(c_{\text{CD45,0}}\right) \quad \text{(S11)}$$

and that of CD2 by:

$$\mu_{\text{CD2}} = k_B T \ln\left(c_{\text{CD2}}/c_{\text{CD2,0}}\right) + \mu^{\text{ex}}_{\text{CD2}}\left(c_{\text{CD2}} + f(c_{\text{CD45}})\right) - \mu^{\text{ex}}_{\text{CD2}}\left(c_{\text{CD2,0}} + f\left(c_{\text{CD45,0}}\right)\right) \quad \text{(S12)}$$

with $\mu_{\text{CD45}}(c_{\text{CD45,0}}) = \mu_{\text{CD2}}(c_{\text{CD2,0}}) = 0$. Thus, the excess chemical potential of CD2 is affected by the excluded area taken up by the accumulated CD45 molecules in the trap, with f being a function depending on the concentration c_{CD45}. The chemical potential is at equilibrium related to the hydrodynamic area and $\varepsilon_{\text{hydro}}$ of the trap by:

$$\mu_{\text{CD45}} = \int_0^{\epsilon_{\text{hydro}}} A_{\text{hydro,CD45}}(c_{\text{CD45}})\, d\epsilon_{\text{hydro}} \quad \text{(S13)}$$

$$\mu_{\text{CD2}} = \frac{A_{\text{hydro,CD2}}(0)}{A_{\text{hydro,CD45}}(0)} \int_0^{\epsilon_{\text{hydro}}} A_{\text{hydro,CD45}}(c_{\text{CD45}})\, d\epsilon_{\text{hydro}} \quad \text{(S14)}$$

where in Supplementary equation (S14) it is assumed that the shielding of the hydrodynamic force on CD2 is dominated by CD45 and varies similarly with concentration as for CD45. Combining these four equations results in:

$$k_B T \ln\left(\frac{c_{\text{CD2}}}{c_{\text{CD2,0}}}\right) + \mu^{\text{ex}}_{\text{CD2}}(c_{\text{CD2}} + c_{\text{CD45}}) - \mu^{\text{ex}}_{\text{CD2}}\left(c_{\text{CD2,0}} + c_{\text{CD45,0}}\right) = \frac{A_{\text{hydro,CD2}}(0)}{A_{\text{hydro,CD45}}(0)} \mu_{\text{CD45}} \quad \text{(S15)}$$

where μ_{CD45} is defined by Supplementary equation (S11) and we as an approximation have set $f(c_{\text{CD45}}) = c_{\text{CD45}}$. The radial concentration profile of CD45 in the double trap was used as input values, $c_{\text{CD2,0}}$ = 610 molecules/μm^2 and $c_{\text{CD45,0}}$ = 140 molecules/μm^2, and the excess chemical potential for CD45 was taken from the fitted curve in Fig. 2B. The excess chemical potential of CD2 was assumed to obey a hard-disk model with radius 5 nm. The values for $A_{\text{hydro,CD2}}$ and

$A_{\mathrm{hydro,CD45}}$ were measured from the double-trap interaction curves to be 419 nm^2 and 1015 nm^2. Solving Supplementary equation (S15) with these parameters gave the curves in Supplementary Fig. S7. The system is assumed to be in quasi equilibrium at all times, which, especially at lower times, is a rather crude approximation, but the obtained curves still capture the essential behaviour of the experimental data.

Supplementary References

1. Jönsson, P. & Jönsson, B. Hydrodynamic Forces on Macromolecules Protruding from Lipid Bilayers Due to External Liquid Flows. *Langmuir* **31,** 12708–12718 (2015).
2. Crocker, J. & Grier, D. Methods of Digital Video Microscopy for Colloidal Studies. *J. Colloid Interface Sci.* **179,** 298–310 (1996).
3. Jönsson, P. *et al.* Hydrodynamic trapping of molecules in lipid bilayers. *Proc. Natl. Acad. Sci. U. S. A.* **109,** 10328–33 (2012).
